# Survey of Technology in Network Security Situation Awareness

**DOI:** 10.3390/s23052608

**Published:** 2023-02-27

**Authors:** Junwei Zhang, Huamin Feng, Biao Liu, Dongmei Zhao

**Affiliations:** 1 School of Cyber Engineering, Xidian University, Xi’an 710126, China; 2 School of Cyber Engineering, Beijing Electronic Science and Technology Institute, Beijing 100070, China; 3 College of Computer and Cyber Security, Hebei Normal University, Shijiazhuang 050025, China

**Keywords:** situation awareness, situation assessment, situation prediction, NSSA visualization, artificial intelligence

## Abstract

Network security situation awareness (NSSA) is an integral part of cybersecurity defense, and it is essential for cybersecurity managers to respond to increasingly sophisticated cyber threats. Different from traditional security measures, NSSA can identify the behavior of various activities in the network and conduct intent understanding and impact assessment from a macro perspective so as to provide reasonable decision support, predicting the development trend of network security. It is a means to analyze the network security quantitatively. Although NSSA has received extensive attention and exploration, there is a lack of comprehensive reviews of the related technologies. This paper presents a state-of-the-art study on NSSA that can help bridge the current research status and future large-scale application. First, the paper provides a concise introduction to NSSA, highlighting its development process. Then, the paper focuses on the research progress of key technologies in recent years. We further discuss the classic use cases of NSSA. Finally, the survey details various challenges and potential research directions related to NSSA.

## 1. Introduction

Recent years have witnessed the rapid development of emerging technologies, such as big data, cloud computing, the Internet of Things (IoT) [1,2], and blockchain [3]. Computer networks have become the supporting infrastructure for informatization construction, profoundly affecting economic development and human lifestyles [4]. Since the current internet infrastructure is witnessing explosive growth in terms of connected devices and the amount of generated content, despite the networks providing various conveniences for people, some security concerns may arise due to potential attacks [5]. Specifically, most network applications have security vulnerabilities, network attack threats are becoming more and more rampant [6], and network security risks are becoming more and more complex. On a global scale, the internet is frequently attacked, such as Sierra Wireless, an IoT solution provider [7], encountering a ransomware company, which damaged its internal system and made its official website inaccessible. The stock price fell 11.95% that day. Although it did not affect the products and services of the company’s other customers, it did affect the company’s products and services, and business development also experienced a certain impact.Moreover, the Portuguese energy giant, Energias De Portugal (EDP), suffered a ransomware attack that saw 10 TB of sensitive corporate data stolen and used to blackmail the corporation for nearly EUR 11 million [8].

In recent years, network attacks have gradually shown large-scale, coordinated, and multi-stage characteristics. Network attacks are no longer isolated incidents, and multi-step attacks are emerging one after another. For example, the increasingly widespread Zeus botnet [9], and worm attacks are highly concealed, penetrating and targeted multi-step attacks [10]. Therefore, it is urgent to study the network security situation awareness (NSSA) for multi-step attacks to improve the identification and recognition of multi-step attacks [11]. The rise of the concept of NSSA has aroused the interest of researchers simultaneously [12,13,14,15,16].

Although there is no uniform definition for NSSA, in general, NSSA extracts the elements which affect the network security, understands, evaluates, and predicts the development trend of the future network. Quantitative analysis and accurate prediction of network security is a means to provide practical decision support for network administrators, to improve the emergency response [17]. With this concept, NSSA can provide various important benefits to network security, as follows:

- The first is to be comprehensive, to perceive the overall situation and all network security events from the perspective of the entire network;
- The second is to be able to accurately and effectively detect network attacks;
- The third is real-time network attacks that break out instantaneously, and real-time detection and real-time evaluation are the core indicators of NSSA.

With these unique advantages, NSSA has become a crucial solution and critical development direction of network security protection since it can change the situation of “active attack by hackers and passive defense by enterprises”. Driven by the recent advances of NSSA, several reviews of related work have appeared. For example, the study in [18] provided a survey on the concept and review of research on CSA. It is worth noting that NSSA and CSA are two different expressions, and different authors use them differently, but both refer to network security situational awareness. The author in [19] presented a literature review of NSSA, based on systematic queries in four leading scientific databases. Moreover, the visualizations to support NSSA were investigated in [20]. An overview on the analysis framework of NSSA and comparison of implementation methods was provided in [21]. Another work in [22] presented a systematic explanation for the definition of NSSA and the understanding of the basic concept. Similarly, the authors in [23] discussed the NSSA concept from the architectural perspective, along with the structure and key technology of NSSA. Furthermore, a survey of prediction, and forecasting methods used in NSSA was proposed in [24]. The comparison of the related works and our paper is summarized in Table 1.

Although NSSA has been studied extensively in the literature, there has been no work to conduct a comprehensive and dedicated review of the NSSA technology. The critical contribution of this paper lies in the extensive discussion of NSSA, including the history, model, and taxonomy. Meanwhile, we start from the three functional modules of situation element acquisition, evaluation, and prediction, and introduce the current research situation of each technology in detail. We further discuss the classic use cases of NSSA. Finally, we discuss several important research challenges and future directions in NSSA.

**Figure 1:**
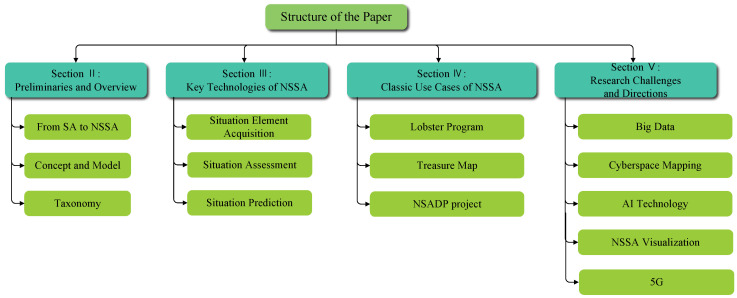
Organization of this survey.

This survey structure is shown in Figure 1. The rest of this survey is outlined as follows. Section 2 introduces the origin, concept, model and the taxonomy of the NSSA. Section 3 discusses the critical technologies of NSSA, including the scientific research literature of the three functional modules in recent years, the technical problems that have been solved, and the technical problems that need to be solved, along with possible directions for future research. Section 4 presents the classic use cases of NSSA. Section 5 concludes the paper and provides an outlook on future research.

## 2. Preliminaries and Overview

The background and history of NSSA are presented in this section. The model and the taxonomy of NSSA are also discussed.

### 2.1. From Situation Awareness (SA) to NSSA

“Situation” was first used in military warfare to describe large-scale research objects’ overall state and changing trends. These research objects are dynamic, affected by many factors, and have relatively complex internal structures. Therefore, a situation is not an illustration of a single situation or state but a comprehensive concept of an entirety that includes a single element.

The early seeds of SA as an area of study were formed in the late 1980s. The foundations of a theory of how people acquired and maintained SA has developed several methods for measuring SA in system design evaluation. The 1990s have expanded this early work to include many other domains and research objectives. From its beginnings in the cockpit realm, more recent work has expanded to include air traffic control, medicine, control rooms, ground transportation, maintenance, space, and education. Research objectives have also grown from one of system design and evaluation to focus on training, selection, and more basic research on the cognitive processes themselves [25].

SA originated from the research of the American military in a military confrontation. In military terms, the goal of situational awareness is to give commanders an understanding of both sides, including the position, current status, and capabilities of the enemy so that they can make quick and correct decisions to know one another. At the International Human Factor Annual Conference, Endsley in [26] first suggested the idea of situational awareness, which is “to identify and grasp environmental aspects in a given location and time, and to predict the future trend”.

Tim Bass [27] of the US Air Force Communications and Information Center proposed NSSA and integrated the concept of SA into the field of cyberspace security for the first time. NSSA is designed to provide network security administrators with a basis for decision making to shorten decision-making time, which can effectively improve the network protection awareness of managers. Franke U. [19] believes that the scope of situational awareness is very large, and NSSA is a part of it, which highlights the “network” environment. However, this definition is not clear enough and does not specify whether it is a safe direction for situational awareness. The research of [22] proposes that NSSA is a series of processes for identifying and understanding the state of network security, which mainly includes three steps: Integrate the original data steps measured from the system and realize the extraction of the background state and activity semantics of the system. Second, identify the various types of network activities that exist and the intentions of abnormal movements in them. Finally, the network security situation characterized by this and the influence of the situation on the normal behavior of the network system is obtained.

Then, the research in [28] used a rough set attribute reduction algorithm to extract core attributes and used a particle swarm optimization algorithm to optimize the radial basis function neural network to identify network attacks. In another study, Ref. [29] divided the network situation level, optimized the back propagation (BP) neural network parameters through the simulated annealing algorithm, and determined the network space situation awareness level.

Jia et al. [23] proposed the definition of NSSA in a large-scale network environment. The specific content is as follows: NSSA is to extract, understand, and evaluate the security elements that affect the network security situation, and predict the future security situation based on the assessment results. Moreover, the research in [30] integrated security information from three dimensions, including threat, vulnerability [31], and stability at the decision-making level to measure the security status of the entire network. Zheng et al. used Dempster–Shafer (D-S) evidence theory to integrate host firewall data, web firewall data, and intrusion detection data to evaluate network security.

To our knowledge, academic research on NSSA has increased in frequency and depth. However, as of this writing, a consistent and thorough definition of NSSA has not been developed. Therefore, the systematic and complete definition of NSSA is also an important topic for future research.

### 2.2. Concept and Model

#### 2.2.1. Model Overview

**Figure 2:**
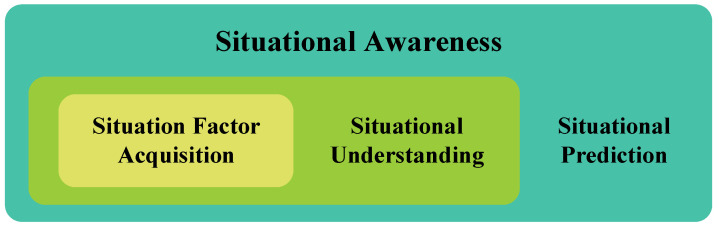
Endsley situational awareness model.

The earliest and most widely used definition of situational awareness is that of Endsley [32]: Perception of the elements in the environment within a volume of time and space, the comprehension of their meaning and the projection of their status in near future. In this definition, situational awareness is divided into three levels as shown in Figure 2.

The multi-level model is the earliest situational awareness model, and it consists of three levels. The first level is situation element acquisition, and the most important task is to obtain critical data. The second is situation understanding, which is responsible for analyzing the critical data obtained by the previous level. The last level is situation prediction [18], which uses the data analysis findings from the level before to forecast what may happen in the future.

Additionally, the JDL model is the traditional SA model [33], a data fusion model proposed by the United States Joint Laboratory (JDL). The SA model is broken down into five levels in this concept. The first level is data preprocessing; the main task is to process incomplete data and remove and filter redundant data information. The second level is event extraction, which carries on the relatively structured data and information already processed at the first level, standardizes network events, and prepares for the next level. The third level is the situation assessment, which evaluates the extracted events to form a comprehensive situation map of the network and provides auxiliary information for the administrator to make decisions. The fourth level is impact assessment, which maps the formed situation to the future environment and evaluates the impact of future battlefields or predicted combat behaviors. The fifth level is resource management, process control, and optimization; the major work is to conduct real-time monitoring and evaluation of the whole data fusion process and integrate all levels of information to achieve the optimization of relevant resources [34].

**Figure 3:**
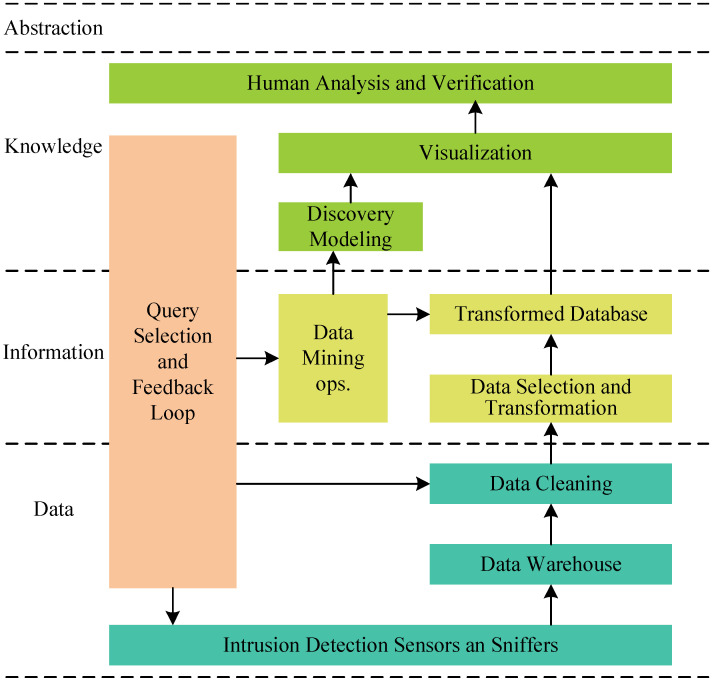
Intrusion detection data fusion model.

Safety is the major focus of situational awareness in network applications. A multisensory-based intrusion detection framework was presented by Tim Bass [35] (see Figure 3). The model is the prototype for situational awareness in network security, and the reasoning framework includes intrusion detection, intrusion behavior, intrusion identity recognition, scenario assessment, threat assessment, etc. The term NSSA was also discussed by Wang [36] according to the Chinese translation of Endsley.

**Figure 4:**
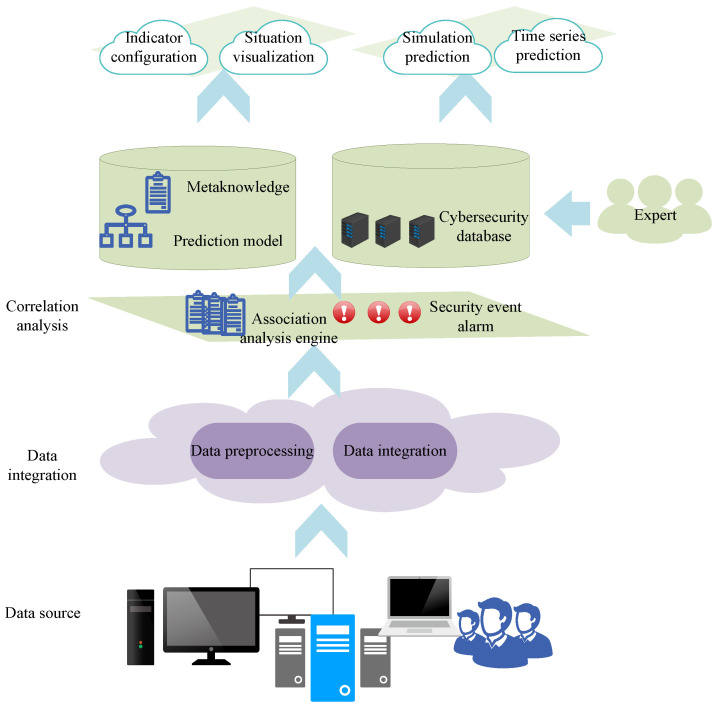
NSSA model for large-scale networks.

Additionally, an NSSA model based on netflow was proposed by Lai et al. [37] in their study. Utilizing netflow technology can effectively achieve NSSA, quickly identify weaknesses and potential threats, and graphically convey them to decision makers for thorough network monitoring. Performance optimization concerns must also be further investigated because the system must handle enormous volumes of data and information. The properties of huge data in large-scale networks, as illustrated in Figure 4, led Jia et al. [38] to develop an NSSA model for such networks.

The NSSA system proposed by Kokkonen T. [39] consists of an input interface layer, an information normalization layer, a data fusion layer and a visualization layer. The model emphasizes the role of visualization, which also includes a human–computer interaction interface and an information-sharing interface. By combining and extending the JDL data fusion model and Endsley’s situation awareness model, Kokkonen [40] proposed an NSSA model, which consists of four layers, including a recognition layer, understanding layer, prediction layer and measure layer from bottom to top. Compared with the three-tier architecture of the traditional model, this model adds a measured layer, which is more comprehensive, by providing alternative measures and their impacts to assist decision makers in making decisions.

Most of the current models are based on the three-tier architecture of the traditional model, supplemented from the perspectives of dynamic circulation, visualization, and automation, and enrich and refine the model according to the needs of different application scenarios.

#### 2.2.2. Explanation of the Example

Here, we present an example of the operational principles and data processing methods of an NSSA model [38] as shown in the figure. The model consists of four levels. The first level is data integration, which involves preprocessing and integrating multi-source data with different formats. Data are integrated into a unified format by deploying agents to the data sources, and then redundant and noisy data are removed. The second level is correlation analysis, which applies association rules in the network security knowledge base to establish reliability-based correlations among different alarm information and match alarm events. The events are analyzed in conjunction with vulnerabilities, assets, and the events themselves to effectively reduce the false-positive rate of security alarms. The third level is the indicator system and situational display, which calculates network security indicators using scientific methods based on the indicator model and correlation analysis results in the knowledge base, and displays the network security situation visually. Specifically, the basic operation index, network vulnerability index, and network threat index are calculated separately and then integrated to obtain the network security index. Define the network security index at time *t* as follows:

(1) $$\left[\begin{array}{c} C(t)\\ I(t)\\ A(t) \end{array}\right]=\left[\begin{array}{cccc} C_{1} & C_{2} & \cdots & C_{n}\\ I_{1} & I_{2} & \cdots & I_{n}\\ A_{1} & A_{2} & \cdots & A_{n} \end{array}\right]\left[\begin{array}{c} E_{1}\left(t\right)\\ E_{2}\left(t\right)\\ \vdots \\ E_{n}\left(t\right) \end{array}\right]$$

In the formula, $E_{i}\left(t\right)$ represents the threat index of security event $E_{i}$ at time *t*, *n* is the number of security event types, and C(t), I(t), and A(t) respectively represent the confidentiality, integrity, and availability indices of *T* at time *t*. The fourth layer is situation prediction. Based on the prediction model learned from historical data, a prediction algorithm based on mean and trend features is used to predict network security events.

### 2.3. Taxonomy

Although considerable work has been conducted on the definitions and associated models of SA and NSSA, little has been conducted to date to classify their constituents. The most representative taxonomy of NSSA is provided by Evesti et al., which includes data collection (actions and policies), analysis, and visualization [41]. What is missing from that taxonomy is a projection-level taxonomy and any associated tools and methods. To overcome the completeness of taxonomy, Martin et al. improved the category [18].

**Figure 5:**
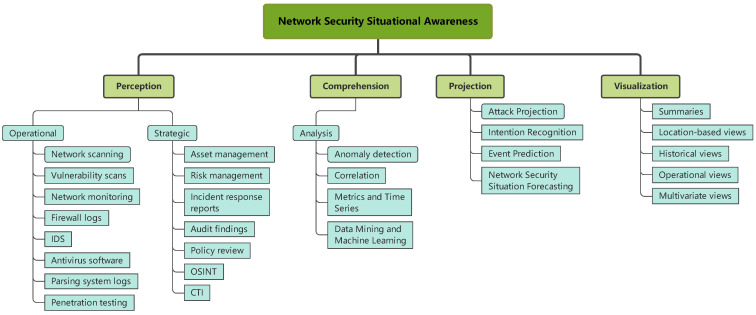
Taxonomy of NSSA tools and components.

This classification adjusts the category to reflect Endsley’s SA three-tier model. Specifically, the perception part mainly uses different tools to obtain network security data, including scanning tools, intrusion detection systems and so on. Comprehension is based on perception, through the calculation and processing of massive data, bypassing complex and difficult appearances, and helping analysts and decision makers understand network status from a higher-dimensional perspective. Projection is based on the perception, comprehension, and processing of historical and current situation data series, through the establishment of mathematical models, exploring the laws of evolution, and reasoning about future development trends and conditions. However, in our opinion, the visualization should be a step after analysis and projection, an essential part of presenting the results of all analysis and projection to administrators, and should not be placed under comprehension. So, the paper moves the visualization to the top level. Figure 5 outlines the improved classification of NSSA, with the most significant changes occurring at the top level, where visualization is considered the final stage of NSSA.

## 3. Key Technologies of NSSA

**Figure 6:**
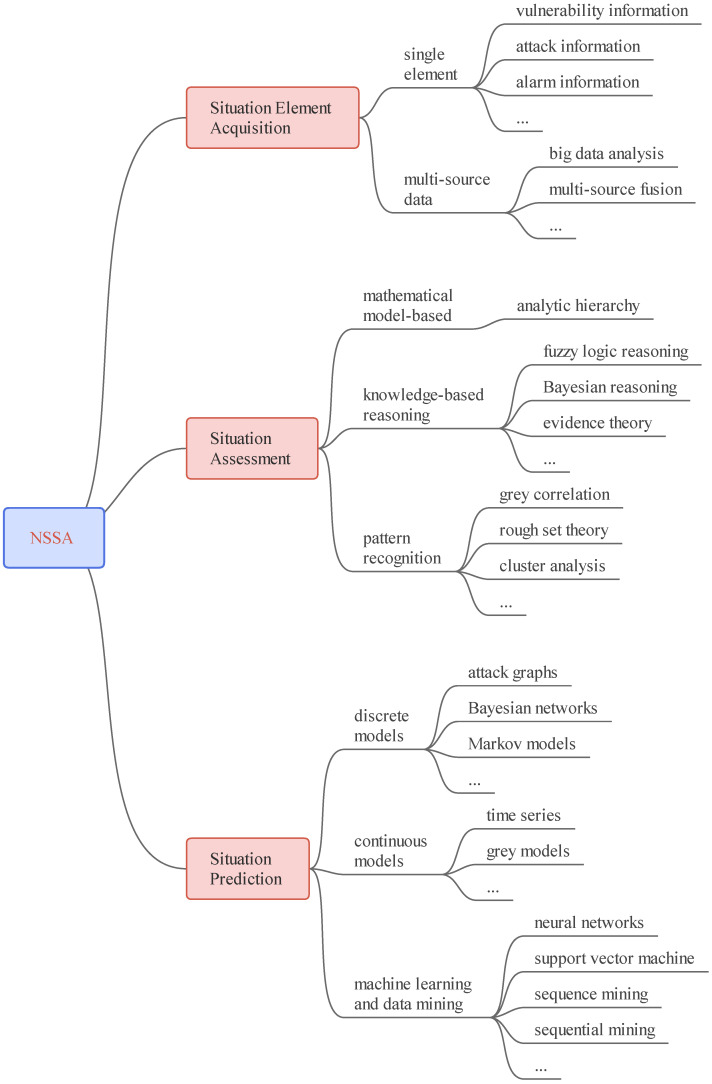
The key technologies of NSSA.

There are still some issues with researchers’ comprehension of the relationship between NSSA in various settings, despite the fact that different researchers have diverse perspectives on how to divide the many stages of NSSA. Researchers most frequently utilize these three functional modules to classify NSSA: situation element acquisition, situation evaluation, and situation prediction. As depicted in Figure 6, this section classifies and introduces the primary NSSA technologies.

### 3.1. Network Security Situation Element Acquisition

Undoubtedly, situation element acquisition is the premise of NSSA. In most cases, the situational elements mainly include the static configuration and dynamic information of the network [42]. The former contains data about the topology of the network, vulnerabilities, and status. The latter phrase alludes to threat data that have been gathered through log gathering and analysis technologies of various defenses. The efficient integration of this information provides the basis for the high-dimensional abstract understanding of situational awareness. Table 2 summarizes the work on the network security situation elements acquisition.

#### 3.1.1. Literature Overview

Researchers mainly extract security data from two levels: single element and multi-source data. Extracting from a single element is mainly used for specific data, such as vulnerability information, warning information, etc., such as the study in [43,44] only gathering network vulnerability information. Barford et al. [47] used attack data and threat information obtained by Honeynet to evaluate the network status, whereas Ning et al. [45,46] merely collected network alarm information and examined the status of the alarm information to assess the danger of the network. The commonality among the aforementioned studies is that they all gather, examine, and research a single network element, which makes it impossible to gain full information, understand the whole situation or react to the complex and dynamic network environment.

With this in mind, many researchers aim to obtain information from multiple sources and comprehensively evaluate the network security situation from multiple perspectives. For example, Wang Juan’s study [48] proposed a layered index model of network security situational awareness based on an index system. The model extracts data from multiple sources of information security following the requirements of hierarchy, information source, and the distinction between structural.

Li et al. proposed a novel multi-source information fusion based heterogeneous network embedding approach [55], for which they jointly modeled the structural proximity, attributed information and labeled information in the framework of non-negative matrix factorization. Additionally, there are many research works on the security extraction of multi-source heterogeneous information network [49,50,51,52,53]. A probabilistic neural network-based technique for extracting security situational elements was suggested by Chang et al. in [54], which addressed the issue of situation element extraction’s poor efficiency and accuracy in complex network environments.

#### 3.1.2. Strengths and Weaknesses Analysis

The survey indicates that the majority of researchers concentrate on the single-element acquisition, and the minority of researchers tend to the acquisition of multi-source information. Information data collected from a single source, local network, or a single level have some restrictions and cannot fully describe the current situation of the network; subsequent state analysis and trend prediction require in-depth correlation analysis of multi-source and omnidirectional data. Consequently, the components of a multi-source extraction are necessary. Multi-source data and information, however, do more than only decrease extraction efficiency. However, the multi-source data collection has a lower extraction efficiency due to the severe inconsistency of manufacturers, standards, and targets in current hardware equipment, software systems, and data sources, and inconsistencies in the collective’s format, dimension, and semantics. In addition, complex operations, such as the cleaning, integration, specification, and transformation of the collected data, are required. Desultorily data also causes problems with information fusion and redundant processing, and therefore, improving the extraction technique is still a popular area of study.

In addition, the existing information network has grown into a vast, complex, nonlinear system with a high degree of flexibility and dynamics. The generation of secure data is fast, large in scale, and complex in format. For limited communication and computing resources, it is necessary to adopt targeted collection methods, such as on-demand collection and segment collection to reduce the requirements for communication and computing resources for information extraction. There are many theoretical and technical problems in current feature extraction [56]. However, at present, the accuracy of detection results is still insufficient, such as redundant data or error alarm information [57], which still has a great influence on the reconstruction of attack activities. The efficiency of detection is not high. For example, many off-line methods are used for correlating analysis and attack process reconstruction, which cannot meet the requirements of rapid response.

### 3.2. Network Security Situation Assessment

A crucial part of NSSA is the network security situation evaluation [58]. A network security condition evaluation incorporates several security data sources. Based on a mathematical model and formal logic, the evaluation value of the current network security situation is derived in compliance with the specific requirements of network security assessment. The evaluation value is similar to the stock index, national index and so on to reflect the security state of the network. The mapping from the situation factor to the situation outcome value is, in essence, what constitutes a network security situation evaluation [59]. In this article, we categorize network security scenario assessment techniques into three groups based on current developments in NSSA: mathematical model-based technique [60], knowledge-based reasoning, and pattern recognition [61]. Table 3 summarizes the work on the network security situation assessment.

It is frequently required to create a network security indication system before performing a network security scenario evaluation. The indicator system is defined as a unified whole composed of a number of interconnected and complementary indicators to evaluate and reflect a certain situation in a certain field. Many scholars have established a network security index system with their own rationality on the premise of a large number of summaries. Wang Juan et al. [48] proposed a layered index model and 25 candidate indicators based on comprehensive security assessment and large-scale network research results and established an index system for situational awareness. On the basis of this achievement, Yue [62] proposed an NSSA system model based on the index system. According to functional requirements, the system is divided into seven modules: “situation data collection-index extraction-index system establishment-data storage-situation assessment-situation prediction-visualization”. It briefly introduces the function of each module and its key technical implementation. The construction of the network security index system is the core of the entire network security situation assessment. Its main goal is to establish the mapping relationship between the situation assessment factor and the final situation value. It must also be improved. Just like the above-mentioned representative index system, it has the characteristics of the stage at that time, so the construction of the index system is a process of dynamic evolution.

**Table 3:** Researchers on the network security situation assessment of relevant work.

Ref.	Approach/Model	Description	Shortcomings
[63]	Analytic hierarchy process (AHP)	Quantitative evaluation at four levels	High time complexity
[64,65,66]	AHP	Hierarchical analysis of multi-source data	High time complexity
[67]	AHP	Multi-layered methodology for situation assessment	Poor real-time performance
[68]	AHP and fuzzy evaluation method	AHP combined with fuzzy evaluation	Low accuracy
[69]	Fuzzy inference model	Generate risk assessment results using fuzzy inference models	Poor real-time performance
[70,71]	Rough set theory	Build decision tables for assessment	Low precision and high time complexity
[72]	Rough set and fuzzy rough set	Mix information processing improves output accuracy	High time complexity
[73]	Deep learning	Adaptive momentum into the training process of the neural network	Over-dependence on parameter selection
[74]	Deep neural network	Combine Deep Autoencoder (DAE) with Deep Neural Network (DNN)	High time complexity

#### 3.2.1. Literature Overview

**Figure 7:**
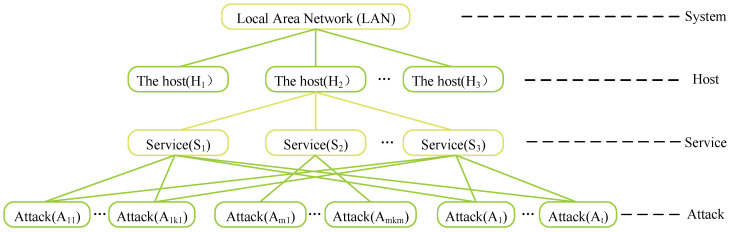
Hierarchical network system security threat situation quantitative assessment model.

The analytic hierarchy process (AHP) is the most common situation assessment method based on mathematical models. The representative research results are the quantitative assessment model of the network system security threat situation proposed by Chen in [63]. The model is divided into four levels from top to bottom—system, host, service, and attack—as shown in Figure 7. However, the model has some shortcomings: only intrusion detection systems (IDSs) alarm information is used in the evaluation method. In real network system deployment, security factors, such as firewalls and system logs, are indispensable. If security information from multiple sources is not included, the situation assessment will be lost. For this reason, the research in [64,65,66] all optimized the above-mentioned hierarchical model, and the purpose of optimization is to make the hierarchical analysis of more sources more accurate. Others, such as Jia [67], suggested a multi-layered methodology for evaluating the security of a network, which can reflect the security state of the information system at a certain stage but also has shortcomings, which is that it cannot analyze the state of network security in real time.

The knowledge-based reasoning method mainly relies on the knowledge and experience of experts in the process of constructing the evaluation model, and analyzes the current network security situation according to the experience of the experts. Common knowledge-based reasoning methods include fuzzy logic reasoning, Bayesian reasoning, and evidence theory. To assess the network security situation, for instance, Kong et al. suggested a fuzzy comprehensive assessment model that combines AHP and the fuzzy evaluation method [68]. Alali et al. proposed to use a fuzzy inference model to generate risk assessment results based on the four risk factors of vulnerability, threat, likelihood and impact, designate the scope of risk that can threaten any entity, and try to address such issues to the proposed entity. Afterward, various analyses of these factors were carried out to verify the feasibility of the method [69]. The grey correlation approach, rough set theory, and cluster analysis method are examples of pattern recognition techniques. Reference [70] provided a detailed analysis of the decision table construction process as applied to the rough set approach of situation appraisal. A mixture of the rough set and the fuzzy rough set was utilized for information processing in reference [72], which increases the accuracy of calculation outputs to address the drawback of accuracy loss when using rough set theory for situational awareness. A network scenario assessment approach based on rough set analysis was developed in reference [71] by fusing conditional attribute reduction and decision rule reduction.

**Figure 8:**
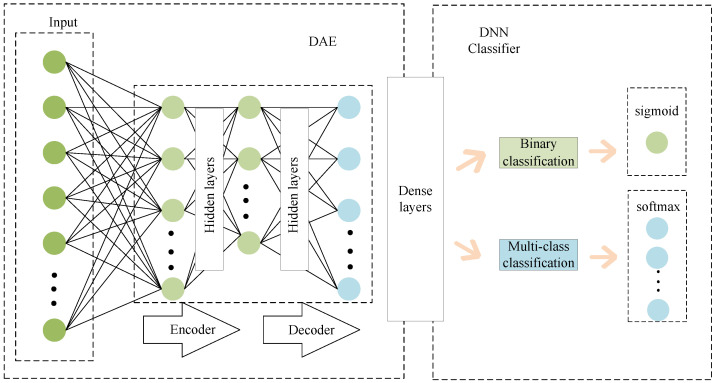
A classification model combining DNN and DAE.

Moreover, because of its powerful learning capabilities, versatility, and broad coverage, deep learning has effectively been implemented in numerous industries, including anomaly detection in medical images [75], target monitoring and recognition [76,77], and feature learning [78]. Therefore, many researchers have recently used deep learning in network situation assessment [73]. For example, the study in [74] proposed a network security situation assessment method based on deep adversarial learning, which establishes a new model that combines deep autoencoder (DAE) with the deep neural network (DNN), as shown in Figure 8. They compared the results of other models to show that the proposed model is more accurate for identifying network attacks and can evaluate the network situation more comprehensively and flexibly.

#### 3.2.2. Strengths and Weaknesses Analysis

Although the knowledge reasoning-based approach to assessing network security has some artificial intelligence (AI), it is hampered by the difficulties of gathering inference rules and previous information. Even though the evidence theory has the benefit of being simple to obtain and integrating a variety of expert knowledge and data sources, when there is conflicting evidence, it is likewise bad to have excessive computational complexity.

The complete network state may be integrated to some extent using conventional methods based on the mathematical logic model and knowledge reasoning model, which also provide network management with decision-making advice. It is difficult to evaluate the situation in light of the network’s real-time state because some traditional methods, which typically rely excessively on expert assessments and logical reasoning, are not equipped to handle the demands of dealing with a large volume of network traffic and attacks as the network enters the big data era [74].

The pattern recognition approach divides situations using pattern matching and mapping by first applying machine learning (ML) to construct a situation template. It is more complex than knowledge reasoning and depends less on specialized information and expertise. The pattern recognition assessment method has the advantages of being highly efficient, having an enormous processing capacity, and not relying too heavily on expert knowledge. The drawback is that it is challenging to deal with increasingly complicated data during the pattern extraction step, which reduces the effectiveness of the evaluation. In addition, fuzzy theory paired with ML can better reflect changes in network state. A fuzzy neural network (FNN) can also be useful in scenario evaluation [79].

### 3.3. Network Security Situation Prediction

The ultimate goal of the assessment is to predict and use historical data to provide a management framework for future network security, making network security management change from passive to active. Network security situation prediction (NSSP) is based on historical information and network security conditions to predict the development trend in the future. It is the highest level of full situational awareness and plays an essential role in network security defense [80].

#### 3.3.1. Literature Overview

Network attacks are random and uncertain, and the change in the security situation is bound to be a complex nonlinear process [81], so traditional prediction models are difficult to apply. In previous studies, researchers classified NSSP methods into the following stages. First, Wei et al. [82] divided NSSP into neural networks, time series forecasting methods, and support vector machine (SVM) methods. Second, Liu et al. surveyed several existing cybersecurity situational prediction techniques and classified them according to their theoretical backgrounds [83], including ML, Markov models [84], and grey theory. Third, Abdlhamed et al. published two classification methods successively. The prediction methods are divided into methods using hidden Markov models, methods based on Bayesian networks, and genetic algorithms in the research [85]. A survey was then released to categorize forecasting methods as well as forecasting systems, arguing that forecasting methods could be based on alert correlations, action sequences, statistical and probabilistic methods, and feature extraction, among others [86].

This section summarizes the research progress of network security situational prediction according to the classification in [24]. It categorizes methods according to the theoretical background on which the forecast is based. Typically, predictive methods in network security use models to represent an attack or network security situation. Table 4 summarizes the researchers’ work on network security situation prediction.

The first category is discrete models, including graph models and game-theoretical models; graph models include attack graphs, Bayesian networks, and Markov models. An attack graph is a graphical representation of an attack scenario introduced in 1998 by Phillips and Swiler [87], which quickly became a popular method of the formal expression of attacks. A technique for creating attack graphs utilizing information from the infrastructure of the maritime supply chain was presented by Nikolaos [88]. This approach provides all potential access points that could be used. A recommender system then foretells how the network will be attacked in the future. The approach in [89] employs a Bayesian network to describe the assault propagation process and extrapolate the likelihood of compromised sensors and actuators. The study in [90] examined the weaknesses of conventional attack prediction algorithms and proposed to set up a hidden Markov model based on the alteration of the host’s security status with the alteration of the observation sequence to more accurately reflect the network security state. To more accurately calculate the projected attack probability and decrease the frequency of false alarms, the parameters of the hidden Markov model (HMM) were improved. Quantitative analysis was performed to determine the security posture across the entire network.

Additionally, a weighted HMM-based technique was presented [91] to predict the security condition of the mobile network to address the problem that traditional HMM based algorithms for predicting network security are not accurate. To overcome the slow data training speed in mobile networks, multiscale entropy was applied, and the parameters of the HMM situation transition matrix were also improved. Game-theoretical methods seek to identify the optimal strategy for the players rather than the most frequent attack progression shown in historical data, in contrast to graphical model-checking approaches. Therefore, game-theoretical approaches appear promising, particularly for forecasting the behavior of sophisticated attackers. For instance, the study in [92] suggested using game theory in opposition to nature to choose the best bid estimate variant.

**Table 4:** Researchers on the network security situation prediction of relevant work.

Ref.	Approach/Model	Description	Shortcomings
[88]	Attack graph	Identify attack paths	High time complexity
[89]	Bayesian models	Infer the probabilities of sensors and actuators to be compromised	Easy to produce overfitting and reduce the prediction accuracy
[93]	Fuzzy Markov chain	Combines historical data with the level of threat, predict the next threat by value using fuzzy Markov chains	Low prediction accuracy
[92]	game theory	Based on the use of game theory against nature to identify the optimal variant of a bid estimate	Algorithmic complexity is too high
[94,95,96,97]	BP neural network	Adjust and optimize parameters in time through continuous learning	Slow convergence and easy to fall into local optimal solution
[98]	Wavelet neural network	Optimized by genetic algorithms	Low prediction accuracy
[99,100]	RBF neural network	Through training the RBF neural network, find out the nonlinear mapping relationship	Low learning accuracy and poor generalization ability
[101]	Cyclic neural network	Based on recurrent neural network with gated recurrent unit	Only valid for data with sequence properties
[102]	SVM	Use mapreduce to perform distributed training on SVMs to improve training speed	too sensitive to parameters
[103]	SVM	Optimize SVM parameters based on grey wolf optimization algorithm	Can’t handle massive data
[104,105,106,107]	deep learning/Stacked Denoising Auto-Encoders (SAE) /association rules mining	Improve prediction accuracy and reduce algorithm complexity	Overfitting of low-dimensional data High complexity of high-dimensional data

The second category is continuous models, including time-series and grey models. Lai [108], for example, developed a prediction model based on gray theory and provided an NSSP technique based on simple weighting and grey theory. Zhang et al. [109] utilized the grey correlation model and grey prediction algorithm as an additional NSSP technique. To forecast network security issues, Deng et al. suggested combining neural networks and gray theory, which also produced positive results [110].

The third category is ML and data mining. ML is gaining popularity in a widely explored field in the research community, and network security is no exception. It contains a large number of methods, such as neural networks and support vector machines. Generally, the BP neural network is a very classic neural network model, combined with the network security situation. Lin et al. [94] proposed an NSSP method based on the BP neural network, and Tang [95] proposed an NSSP method based on the dynamic covariance BP neural network. Zhang et al. proposed a network security situation prediction algorithm based on the BP neural network. By adjusting the weights and thresholds, Zhang et al. [97] compared the actual output value of the network with the expected value, and they proposed an NSSP method based on the optimized BP neural network. Previous studies have demonstrated that the BP neural network’s slower convergence speed is a limitation. As a result, it is prone to fluctuation during the learning process and to settle into the best local answer. To improve the network security condition’s forecasting accuracy, the research in [98] created a parametric optimized wavelet neural NSSP model utilizing an upgraded niche genetic algorithm. The radial basis function (RBF) neural network can approximate any nonlinear function with arbitrary precision and is capable of global approximation. A generalized RBF neural network-based approach to network security situation prediction is proposed in order to address the issue of prediction accuracy in network situational awareness [111]. Simulation studies demonstrate that this strategy may more precisely predict situations and enhance network security through active security protection. In addition, the study in [112] optimized the RBF neural network with the hybrid hierarchy genetic algorithm and the simulated annealing (SA) technique.

Furthermore, Feng et al. [101] introduced an NSSP method based on cyclic neural networks in their paper. For the first time, this technique extracts internal and external information features from the initial time-series network data. The deep recurrent neural network (RNN) model is then trained and validated using the extracted features. The well-trained model will produce accurate NSSA predictions after iteration and optimization, and the model is stable for erratic network data.

According to the theoretical basis of SVM, the security situation prediction method based on SVM is very sensitive to the selection of parameters, and the prediction result depends on whether the parameter selection is reasonable. At present, various parameter optimization algorithms are usually used to optimize the model parameters. Hu et al. proposed a MapReduce–support vector machine (MR-SVM) model based on the big data processing framework MapReduce and SVM in 2019, using the cuckoo search algorithm to optimize the SVM parameters and using MapReduce to train the SVM model in parallel, improving the model training accuracy and reducing the training time cost [102]. In the same year, Lu et al. established a kind of NSSP model, which makes it more generalized, and also effectively improves the prediction effect of SVM [103].

The fourth category contains methods that are very specific or difficult to classify. There are many medium prediction methods that will not be introduced one by one here [104,105,106,107].

#### 3.3.2. Strengths and Weaknesses Analysis

Generally speaking, each prediction method has its advantages and limitations. The outstanding self-learning and adaptive capabilities of ML can offer quick convergence and great fault tolerance. To acquire parameters, however, there must be enough training data, and creating neurons that are self-learning and adaptable is challenging. Even though the Markov model may predict different time series, it still requires a set of training data. Additionally, especially in large networks, it is very hard to distinguish all potential states and their transitions. In the short-term prediction, grey theory can offer a sparse sample of data, improving prediction without any training. However, the number of network samples is large and complex, so the limitations of grey theory are also evident. Compared with neural networks, SVM has many advantages, such as strong generalization ability, good adaptability, fast convergence speed, and strong mathematical theory support. It is an excellent security situation prediction algorithm at present.

## 4. Classic Use Cases of NSSA

Because network security is directly related to national security, NSSA has been incorporated into the cybersecurity strategies of many countries. In this section, we will cover some classic use cases of NSSA.

### 4.1. Lobster Program

The full name of the Lobster Program [113] is large-scale monitoring of broadband internet infrastructures. The program was undertaken by the Hellenic Research and Technology Foundation, in conjunction with Alcatel, Symantec, Greek Telecom, Czech National Education and Research Network, European Research and Education Network Association, Vrije Universiteit Amsterdam, and other companies and institutions and schools, aiming at European establishment of a passive monitoring infrastructure for internet traffic, improving the monitoring capabilities of the basic internet, providing early warnings for security incidents, and providing accurate and meaningful performance measurement methods to improve the performance of the internet and the ability to deal with security issues. The Lobster Project lasted more than three years, from January 2004 to June 2007. Its functions include monitoring network performance and availability, which can be directly or indirectly applied to NSSA as core supporting technologies. Although the project has been phased out, the original relevant participants and later service beneficiaries continue their respective research and application work based on this plan. The essential purpose of this plan is to perceive the situation of the network, especially the security situation.

### 4.2. Treasure Map

The National Security Agency (NSA) deployed the Deep Network Surveillance Program also known as the Treasure Map Program in 2011. The research goal of this program is to dynamically incorporate all devices in the entire network into monitoring at any location and at any time to achieve a quasi-real-time, interactive global internet map. In other words, the main task of this plan is network situational awareness. Users of this system include the U.S. National Security Agency, the U.S. Department of Defense, and the Five Eyes Alliance (FVEY), which consists of intelligence agencies in the United States, the United Kingdom, Australia, Canada, and New Zealand. The intelligence and espionage alliance formed by these five countries realizes the interconnection and exchange of intelligence information.

### 4.3. NSADP Project

The British Defense Science and Technology Laboratory (DSTL) and the British Mood company jointly launched the “Network Situational Awareness, Display, and Prediction (NSADP)” project. Through network data collection, analysis, and security situational awareness, the program utilizes a causal modeling approach to support military commanders in taking appropriate proactive actions to respond to adversary cyberattacks.

Except for the above few typical cases, many others have not been introduced one by one, including the Centaur system of the US Department of Defense, the US Eyesight System, the EU’s Wombat Program, the UK Shared Network Security Information Platform, etc.

In short, building an NSSA system aims to achieve active defense against attacks. Many existing critical technical difficulties still need to be further broken through, such as how to accurately and efficiently predict the development trend of the situation, how to judge the attacker’s intention, etc. The breakthrough of difficult points will be essential to realizing active defense.

## 5. Research Challenges and Directions

NSSA is a popular area of study. There are numerous open research fields with significant obstacles that require sophisticated approaches to overcome. New solutions must adhere to a set of constraints and requirements, such as low complexity and reliability. Several possible research directions for these challenges are also discussed.

### 5.1. Big Data

Situational awareness may dynamically reflect the state of network security as a whole and forecast its future course. However, the complexity of the network environment is rising, and the variety of data types and formats is expanding quickly. Massive security data cannot be used directly as an analysis item for determining how secure a network is. Consequently, the use of big data technology opens up possibilities for innovations in extensive network security situational awareness research. Researchers have provided some of the new solutions for this topic [114,115]. A future work proposed in [116] can be improved the recognition rate and reduce the error rate. According to [116], the scheme can seamlessly integrate fuzzy cluster-based association analysis, game theory, and reinforcement learning. Finally, network situational assessment and situational security prediction can be realized. Additionally, several academic studies [117,118,119] demonstrated how big data’s enormous storage, parallel processing, and fusion analysis can help with the NSSA research challenges. Big data technology’s debut presents a chance for big advances in this area. However, the big data-based approach for NSSA still requires a lot of work and careful consideration.

### 5.2. Cyberspace Mapping

To realize an accurate, real-time, and intelligent NSSA system, the first thing to do is to understand the network, which is impossible without cyberspace mapping [120] technology. The application of situational awareness technology is to establish an “immune” system in cyberspace, through all-weather and all-round awareness of cyber threats, especially for deep-level threats that are difficult to detect and defend against traditional security equipment.

In this way, it is possible to respond promptly, deal with it on time, achieve maximum stop loss, eliminate the impact as quickly as possible, carry out necessary countermeasures as needed, break the enemy at the source, and realize the transformation from passive defense to active defense.

Therefore, cyberspace mapping technology is the first link of the network situational awareness system, and it is also essential data support in the cyberspace situational awareness system [121]. In the network situational awareness system, comprehensive and multi-dimensional network asset mapping is indispensable. In today’s country, the concept of cyberspace security has been elevated to a critical level, and it is even more important.

### 5.3. AI Technology

The paper [122] discovered that the majority of the suggested ways are realized through the transformation of the fundamental AI techniques by summarizing papers about AI in network security. These fundamental techniques serve as the cornerstone and demonstrate the viability and superiority of cyber security solutions. To achieve network situational awareness, for instance, Zhao [29] developed a wavelet neural network (WNN) based on a particle swarm algorithm. The study in [123] used the RBF neural network to accurately quantify the network security situation to predict the power information network security situation. Yang et al. [74] established the deep autoencoder-deep neural network (AEDNN) model based on DAE and DNN to offer an NSSA approach based on DNN. By conducting comparative experiments, they demonstrated that the proposed model can improve the ability to identify network attacks. On the other hand, changing only one pixel of the image [124] or just a few bytes in the sample [125] can cause the neural network to misclassify. Furthermore, edge intelligence emerged as a promising solution to leverage massive data distributed at the network edge for training various machine learning models at the edge server [126]. As a “double-edged sword”, AI technology has shortcomings and good performance. Once the information is “infected”, the AI system can be easily deceived, leaving the network in an insecure state. Moreover, the AI models consume more time because they need huge data to complete the training. Therefore, a future research topic is how to use AI technology to improve network security situational awareness while further overcoming its shortcomings.

### 5.4. NSSA Visualization

Franke et al. [19] specifically highlighted the need for going beyond technical aspects of the visualizations to obtain a more comprehensive understanding of NSSA. Although various visualizations have been proposed to support NSSA, there is no clear understanding of the different stakeholders for those visualizations, different types of information visualized, data sources employed, visualization techniques used, levels of NSSA that can be achieved, and the maturity levels of the visualizations, challenges, and practices for NSSA visualizations. Due to the heterogeneity and complexity of network security data, often with multidimensional attributes, sophisticated visualization techniques are needed to achieve NSSA [127]. On this issue, Tamassia et al. [128] provided a crystal-clear statistical finding. The analysis procedure and data in IDS were successfully filtered by Beaver et al. [129], who then visually presented them to administrators. NSSA visualization can be portrayed in two ways [130], emphasizing both interactivity and visualization. However, the most recent work just presents the raw data from real-time data without any analysis, instead emphasizing the cooperative interaction between humans and technology. The flexible analysis of network security situational awareness in general settings still has a long way to go.

### 5.5. 5G

New technologies will bring new security problems, which may be the security problems existing in the technology itself, or the technology may cause other security problems [131]. Since risks can have serious repercussions, security has emerged as the top priority in many telecommunications sectors today [132]. Confidential information will transit at all layers in the future wireless system as the 5G network’s core, and enabling technologies will be included [133,134]. As a result, modern security attacks have become more sophisticated and powerful, making it more difficult to identify them and stop their sabotage.

## 6. Conclusions

This paper presents a state-of-the-art study on the NSSA that can help bridge the current research status and future large-scale application. We first discussed the history of the NSSA. Subsequently, we provided a brief overview of the model and concept of NSSA and introduced the most impactful NSSA models. Then, we combined the previous classification and Endsley’s three-layer model and proposed a new method for the taxonomy of NSSA to overcome the taxonomy issues. Meanwhile, the paper summarized the research progress of NSSA in recent years. It analyzed in detail the critical technologies of situation element acquisition, situation assessment, and situation prediction of three functional modules. We also showed several examples of each technology, illustrating the broad interest in the topic.

The research on NSSA is of great significance to the field of information security. As a branch of computer research that started relatively late, there are still many problems to be solved. The Internet of Things technology and cloud computing technology related to situational awareness are still in their infancy, so mass data acquisition and the high-speed processing technology need to be further improved, and the artificial intelligence machine learning method combined with neural networks and deep learning needs to be further integrated. Moreover, security visualization is a very young term; however, as the number of security-related events generated in modern networks is on the rise, the need for network security visualization systems is felt more than ever.

Even though NSSA is still in its infancy, it will continue to thrive and will be an active and essential research area in the foreseeable future. We believe that this survey will stimulate more attention in this emerging area and encourage more research efforts to absolve the existing technical deficiencies.

## Figures and Tables

**Table 1:** Existing surveys on NSSA topics and our new contributions.

Related Works	Key Contributions	Limitations
[18]	A survey on concept and review of research on cyber situation awareness (CSA)	The applications have not been presented.
[19]	A review of CSA, based on systematic queries in four leading scientific databases	The paper only analyzes the research agenda in the area of CSA.
[20]	A survey on the scientific literature on CSA visualizations	The paper only focuses on visualizations.
[21]	A survey on the analysis framework of Network Security Situation Awareness (NSSA) and comparison of implementation methods	The use of NSSA and applications have not been presented.
[22]	A systematic explanation for the definition of NSSA and the understanding of the basic concept	The paper only focuses on discussing the concept and the framework of NSSA.
[23]	A survey on concept, structure and the key technology of NSSA	The analysis of NSSA technologies is limited. Moreover, discussions for use cases are lacking.
[24]	A survey of forecasting methods for NSSA	The paper only focuses on prediction of NSSA, comprehension and assessment are not considered.
Our paper	An extensive survey on the NSSA integration. First, we extensively discuss the concept and the history of NSSA in network security. Second, the critical research works of NSSA technology are also analyzed in detail, including technical classification, technical characteristics, strengths and weaknesses. Third, the classic use cases of NSSA are provided at the national level. Finally, research challenges and directions are also highlighted.	

**Table 2:** Network security situation elements acquisition.

Ref.	Description	Approach	Strengths	Weaknesses
[43,44]	Obtain vulnerability information	Topological vulnerability analysis (TVA)/attack graphs	Low evaluation difficulty and high evaluation efficiency	The overall security situation cannot be obtained
[45,46]	Obtain alarm information	Intrusion Detection Systems (IDS) /correlation analysis	Low evaluation difficulty and high evaluation efficiency	The overall security situation cannot be obtained
[47]	Obtain attack information	Honeynets	Low evaluation difficulty and high evaluation efficiency	The overall security situation cannot be obtained
[48]	Obtain multi-source information security data	Index system	Perceive the overall network security situation	High evaluation difficulty and low evaluation efficiency
[49]	Obtain the complex network security information	Botnet detection technology	Perceive the whole network security situation	High time complexity
[50,51,52,53]	Obtain multi-source data and information	Multi-source fusion	Perceive the overall network security situation	Reduce the extraction efficiency
[54]	Extract the situation element from multi-source information	Probabilistic neural network	Reduce the system complexity	High time complexity

## Data Availability

Not applicable.

## Abbreviations

The following abbreviations are used in this manuscript:

AI	Artificial Intelligence
AVS	Antivirus Software
BP	Back Propagation
CSA	Cyber Situation Awareness
DAE	Deep Autoencoder
DNN	Deep Neural Network
D-S	Dempster–Shafer
FNN	Fuzzy Neural Network
HMM	Hidden Markov Models
IDS	Intrusion Detection Systems
IoT	Internet of Things
JDL	Joint Directors of Laboratories
ML	Machine learning
NSSA	Network Security Situation Awareness
NSSP	Trusted Execution Environments
RBF	Radial Basis Function
RNN	Recurrent Neural Network
SA	Situation Awareness
SVM	Support Vector Machine
WNN	Wavelet Neural Network
